# Prediction of loss to follow-up in long-term supportive periodontal therapy in patients with chronic periodontitis

**DOI:** 10.1371/journal.pone.0192221

**Published:** 2018-02-08

**Authors:** Di Wu, Hai Jing Yang, Yan Zhang, Xiu E. Li, Yu Rong Jia, Chun Mei Wang

**Affiliations:** 1 Department of Nursing, Peking University School and Hospital of Stomatology & National Engineering Laboratory for Digital and Material Technology of Stomatology & Beijing Key Laboratory of Digital Stomatology, Beijing, China; 2 School of Nursing, Tianjin Medical University, Tianjin, China; 3 Department of Periodontology, School of Stomatology, Tianjin Medical University, Tianjin, China; The Ohio State University, UNITED STATES

## Abstract

**Aim:**

This study examined the predictors of loss to follow-up in long-term supportive periodontal therapy in patients with chronic periodontitis.

**Methods:**

A total of 280 patients with moderate to severe chronic periodontitis in a tertiary care hospital in China were investigated and followed over the course of study. Questionnaires on clinical and demographic characteristics, self-efficacy for oral self-care and dental fear at baseline were completed. Participants were followed to determine whether they could adhere to long-term supportive periodontal therapy. Binary logistic regression analysis was used to examine the association between clinical and demographic characteristics, self-efficacy for oral self-care, dental fear and loss to follow-up in long-term supportive periodontal therapy.

**Results:**

The loss to follow-up in long-term supportive periodontal therapy was significantly associated with age [adjusted OR = 1.042, 95% confidence interval (CI): 1.012–1.074, *p* = 0.006], severe periodontitis [adjusted OR = 4.892, 95%CI: 2.280–10.499, *p*<0.001], periodontal surgery [adjusted OR = 11.334, 95% CI: 2.235–57.472, *p* = 0.003], and middle and low-scoring of self-efficacy scale for self-care groups. The adjusted ORs of loss to follow-up for the middle- (54–59) and low-scoring groups (15–53) were 71.899 (95%CI: 23.926–216.062, *p*<0.001) and 4.800 (95% CI: 2.263–10.182, *p*<0.001), respectively, compared with the high-scoring SESS group (60–75).

**Conclusion:**

Age, severity of periodontitis, periodontal surgery and the level of self-efficacy for self-care may be effective predictors of loss to follow-up in long-term supportive periodontal therapy in patients with chronic periodontitis.

## Introduction

Chronic periodontitis was the primary cause for adult tooth extraction in a report from the third national oral epidemiology investigation in China[1]. Chronic periodontitis is significantly related to oral health, such as halitosis[2]. Supportive periodontal therapy is a lifelong task for patients with chronic periodontitis. These patients must maintain a long-term curative effect by attending regular and frequent follow-ups after completion of the initial therapy[3]. Supportive periodontal therapy is an essential part of integrated periodontal therapy, which includes an update of medical and dental histories; radiographic review; extra- and intra-oral soft tissue examination; dental and periodontal examinations; reexamination of the removal of bacterial plaque, scaling and root debridement; removal of bacterial plaque from sulcular and pocket areas; and polishing the teeth[4]. A previously published study suggested that most patients in developing countries hardly attended dental consultations until obvious symptoms, such as odontalgia and discomfort, were noted because of a lack of awareness for oral self-care[5]. Therefore, these patients often missed the optimal timing for dental visits. The prevalence of moderate to severe periodontitis is increasing.

Initial periodontal therapy effectively removes bacterial plaque and relieves symptoms. However, long-term periodontal health maintenance largely depends on regular and frequent check-ups. Therefore, whether patients enter into supportive periodontal therapy and adhere to long-term follow-ups after completion of the initial therapy is closely related to the clinical outcome of periodontitis. Unfortunately, a considerable number of patients are lost during long-term supportive periodontal therapy in clinical practice despite dentists repeated emphasis on the importance and necessity of follow-up in long-term supportive periodontal therapy and encouragement to enter supportive periodontal therapy and insistence on regular follow-ups.

Bandura found that self-efficacy, as a core concept of social cognitive theory, played a key role in individual action-taking and emotion responding[6]. Self-efficacy theory is widely used in education, clinical psychology, health psychology and other fields[6]. Self-efficacy in clinical practice reflects how confident a patient is in his/her ability to take action to improve their symptoms and maintain their health. People with low self-efficacy for medical compliance may view their tasks or behaviors as difficulties to be solved, even if these tasks are not complicated[7]. Several studies found that self-efficacy contributed to behavioral changes and elimination of bad habits[8–11].

Application of self-efficacy theory in the oral hygiene field was reported in several studies. Earlier evidence demonstrated positive associations between self-efficacy and improved clinical indexes and self-care behaviors, such as brushing and flossing frequency[12–15]. Oral hygiene-related self-efficacy was confirmed as an influencing factor of oral hygiene behaviors, and it predicted clinical outcomes of oral hygiene[9]. Kakudate et al. (2007) developed a task-specific self-efficacy scale for self-care (SESS) and used it in patients with periodontal disease in Japan. They found that oral health care-specific self-efficacy effectively predicted patient’s completion of periodontal treatment and loss to follow-up in all phases of periodontal therapy[7,16]. However, some limitations and factors were also noteworthy. Patients with severe chronic periodontal disease were not enrolled in their study, and several variables, such as educational and socioeconomic status, were not investigated[16].

China is the most populous developing country in the world. The third national oral health epidemiological survey in China reported 38.9% and 71.3% of patients with ≥4 mm clinical attachment loss (CAL) in age brackets 35–44 and 65–74 years old, respectively[1]. Existing studies on patient compliance with periodontal therapy in China demonstrated that patients’ cognitive level of periodontitis was the most important factor affecting their compliance with periodontal therapy[17]. The current status of patient compliance with periodontal therapy and the relationship between self-efficacy and loss to follow-up has not been reported. Therefore, the present study assessed the status of patient compliance with long-term supportive periodontal therapy and examined possible variables to predict loss to follow-up in long-term supportive periodontal therapy in patients with chronic periodontitis.

## Methods

A prospective cohort study was performed from November 2014 to December 2016. Ethical approval was obtained from the Tianjin Medical University Ethics Committee prior to the start of the study (No. TMUHMEC2014001).

### Ethical principles

Participants retained the right to decide their continued participation in this study at all times. The researchers promised that all information related to the participants would not be disclosed to ensure the safety of participants’ privacy. All patients provided informed consent prior to participation.

### Participants

This study recruited 280 subjects with moderate to severe chronic periodontitis at a tertiary grade hospital of stomatology between November 2014 and January 2015 in China. Sample size requirements were determined using logistic regression. The sample size should be 5–10 times the number of independent variables, and our study included 21 independent variables. The calculated sample size ranged from 126 to 252 subjects considering a loss to follow up rate of 20%. Therefore, we recruited 280 subjects into the study[18].

Periodontitis was classified according to the American Academy of Periodontology (AAP) and the Centers for Disease Control and Prevention (CDC). Mild periodontitis was defined as ≥2 interproximal sites with CAL ≥3 mm (not on the same tooth) and ≥2 interproximal sites with PD ≥4 mm (not on the same tooth) or ≥1 interproximal site with PD ≥5 mm. Moderate periodontitis was defined as ≥2 interproximal sites with CAL ≥4 mm (not on the same tooth) or ≥2 interproximal sites with PD ≥5 mm (not on the same tooth). Severe periodontitis was defined as ≥2 interproximal sites CAL ≥6 (not on the same tooth) and ≥1 interproximal sites with PD ≥5 mm[19].

### Exclusion criteria

Patients were excluded if they exhibited physical limitations influencing manual dexterity; had diabetes mellitus, immunodeficiency, or fewer than 20 teeth; or were taking medications that affect the development of inflammation of periodontal tissues. Patients who required prophylactic antibiotic premedication, had undergone extensive non-surgical periodontal treatment within the previous 6 months and periodontal surgery within the previous 2 years or were undergoing any active periodontal treatment were also excluded[7,20].

### Study groups

Participants were classified into two groups according to the outcome loss to follow-up in long-term supportive periodontal therapy. Group 1 included 160 patients who were lost during follow-up periodontal treatment. These patients completed the initial therapy but failed to enter supportive periodontal therapy or entered into supportive periodontal therapy but were lost in long-term periodontal therapy. Group 2 included 120 patients who received periodontal therapy for over 2 years.

### Study protocol

An investigator-training meeting was held prior to the start of the study to confirm the entire research process and unify operative standards. Investigators first used a uniform description to explain the purpose and significance of the study to ensure that all of the subjects provided informed consent to participate in the research. Investigators provided concise instructions on the method and announcements before the patients independently completed the questionnaire. Investigators immediately clarified their explanations if the patient had concerns or questions to ensure that all patients understand and completed the questionnaire fully and correctly. Two investigators immediately confirmed the completeness and validity of the questionnaire to ensure its validity and eligibility.

Clinical parameters were obtained from periodontal examinations performed by one dentist at baseline. This clinician was 30 years old with an MD in stomatology and more than 6 years of clinical periodontitis diagnosis and treatment experience. All patients were given tooth-brushing instructions that included the Bass method[21] and instructions on the use of floss and an interdental brush (JIA). Discussion between the dentist and patients determined the date of the subsequent visit. The outcome of patient loss during long-term supportive periodontal therapy was recorded.

### Demographic and clinical characteristics measurement

Patients’ demographic data and clinical parameters, probing depth, clinical attachment loss, the distance from gingival margin to cementoenamel junction and numbers of teeth present were recorded during the initial examination at baseline. One dentist measured probing depth, clinical attachment loss and the distance from gingival margin to cementoenamel junction at six sites per tooth using a Williams probe that rounded to the nearest millimeter. Probing depth was the distance from the gingival margin to the bottom of the pocket. Clinical attachment loss was the distance from the bottom of the pocket to the cementoenamel junction. We also measured furcation involvement using a Glickman index value but did not incorporate this measurement into our study.

### Assessment of self-efficacy

Kakudate et al. developed a task-specific SESS and used it in patients with periodontal disease. SESS is composed of three subscales: self-efficacy for dentist consultations (SE-DC, five items), self-efficacy for brushing teeth (SE-B, five items) and self-efficacy for dietary habits (SE-DH, five items)[7,22]. A five-point Likert scale from 1 (not confident) to 5 (completely confident) was provided for subjects to choose the most appropriate level. The SESS score for each subject is expressed as the sum of scores for the 15 items. Therefore, SESS scores ranged from 15 to 75. A previous study verified SESS reliability for internal consistency (Cronbach’s α = 0.86) and test-retest stability (Spearman rank correlation coefficient r = 0.73; *p*<0.001) in Japan.

Di Wu et al. (2013) translated SESS into Chinese, amended it to consider the cultural background in China, and re-translated it into English to ensure a valid translation[23]. The revised questionnaire was used on 300 periodontal disease patients to verify its reliability and validity. Test-retest stability (r = 0.922) and internal consistency (Cronbach’s α = 0.88) were observed. Factor analysis extracted three common factors that explained 60.15% of the variance of the total scale, and each item had a high factor-loading quantity (>0.4). The SESS Chinese version exhibited satisfactory validity and reliability.

### Assessment of loss to follow-up

Loss to follow-up was defined if the patient did not present him- or herself at an appointment and did not express a desire to receive consultation within 1 month from the day of the appointment[16].

### Statistical analysis

Statistical analyses were performed using SPSS statistical software (Version 17.0; SPSS, Inc., Chicago, IL, USA). The Kolmogorov-Smirnov test was used to verify the normality of all variables. The chi-square test was used to examine the statistical significance of gender, educational level, monthly family income, tooth number group, severity of periodontitis, periodontal surgery, and the level of self-efficacy for self-care between the two groups. Independent sample t test was used to detect the statistical significance of age, probing depth, clinical attachment loss and SESS scores between the two groups. The Mann-Whitney U test was used to analyze differences in dental fear scale score, self-efficacy for dentist consultations subscale score, self-efficacy for brushing the teeth subscale score, and self-efficacy for dietary habits subscale score between the two groups. Binary logistic regression analysis was used to assess associations between clinical and demographic characteristics, self-efficacy for self-care and patient’s loss to follow-up in long-term supportive periodontal therapy. Variables that exhibited a significant association between group 1 and group 2 were used as independent variables, and loss to follow-up in long-term supportive periodontal therapy was the dependent variable. The results are presented as crude and adjusted ORs with 95%CIs. P-values <0.05 indicated significance.

## Results

### Normality of demographic and clinical parameters

Age, clinical attachment loss and SESS were normally distributed. Probing depth, DFS score, self-efficacy for dentist consultations subscale score, self-efficacy for brushing the teeth subscale score, and self-efficacy for dietary habits subscale score exhibited a skewed distribution.

### Demographic and clinical characteristics of participants

A total of 160 men and 120 women were recruited into the study. A total of 160 subjects failed to enter into supportive periodontal therapy (Group 1), and 120 participants attended regular check-ups and entered into supportive periodontal therapy (Group 2).

### Association between loss to follow-up and clinical and demographic characteristics

Table 1 shows the demographic and clinical characteristics of the two groups at baseline. The mean age of patients in group 1 was significantly higher than group 2 (*p* = 0.037). The educational level of the patients in group 2 was higher than group 1 (*p* = 0.032). The severity of periodontal disease of the patients in group 2 was more serious than the patients in group 1 (*p* = 0.001). More patients in group 2 underwent with periodontal surgery than group 2 (*p* = 0.001). No significant associations in gender, monthly family income, number of teeth present, probing depth, clinical attachment loss, or DFS score were observed in either group.

**Table 1:** Comparison of demographic and clinical characteristics of the two groups at baseline (N = 280).

Variables	Loss to follow-up	Not loss to follow-up	*P* Value
Age (years)	46.42±12.98*	43.14±12.86*	**0.037**^a^
Gender			
Male	95	65	
Female	65	55	0.396^b^
Educational level			
Associate degree education and lower	36	15	
Baccalaureate education and higher	124	105	**0.032**^b^
Income of family per month(yuan)			
<3000	24	12	
3000–6000	73	48	
≥6000	68	55	0.620^b^
Number of teeth present			
18–23	12	10	
24–26	39	25	
27–32	109	85	0.775^b^
Severity of periodontitis			
Moderate	86	41	
Severe	74	79	**0.001**^b^
Periodontal surgery			
Surgery	6	28	
No surgery	154	92	**0.001**^b^
PD (mm)	3.32 (2.98–3.88)^§^	3.59 (2.97–4.10)^§^	0.170^c^
AL (mm)	4.03±0.84*	4.22±0.96*	0.068^a^
DFS score	37.00 (30.00–49.75)^§^	32.50 (27.25–45.00)^§^	0.093^c^

Bolded values indicate statistical significance.

^a^ P value based on independent sample t test

^b^ P value based on the chi-square test of independence

^c^ P value based on the Mann-Whitney U test

* Mean±SD

^§^ median (IQR, Q1-Q3)

### Self-efficacy of participants

Tertile cutoff points for the low-, middle-, and high-scoring SESS groups were 15–53, 54–59, and 60–75, respectively. The mean SESS score and standard deviation (SD) of all participants were 57.03±9.08. The mean SESS score and SD of group 1 was 52.69±8.04, which indicated a middle-scoring SESS group. The mean SESS score and SD of group 2 was 62.83±6.91, which indicated a high-scoring SESS group.

### Association between loss to follow-up and self-efficacy

Table 2 shows the self- efficacy of the two groups at baseline. The numbers of participants failing to enter into supportive periodontal therapy for the low-, middle-, and high-scoring SESS groups were 86, 49, and 25, respectively. SESS score (*p*<0.001), SESS score group (*p*<0.001), self-efficacy for dentist consultations subscale score (*p*<0.001), self-efficacy for brushing the teeth subscale score (*p*<0.001), and self-efficacy for dietary habits subscale score (*p*<0.001) significantly correlated with loss to follow-up.

**Table 2:** Comparison of self-efficacy in the two groups at baseline (N = 280).

	Did not enter SPT	Entered SPT	*P* Value
SESS score	52.69±8.04*	62.83±6.91*	**<0.001**^a^
SESS score group			
15–53 (low)	86	7	
54–59 (middle)	49	29	
60–75 (high)	25	84	**<0.001**^b^
SE-DC	18.00 (17.00–20.00)^§^	21.18±2.78*	**<0.001**^c^
SE-B	16.44±3.77*	20.00 (18.25–23.00)^§^	**<0.001**^c^
SE-DH	19.00 (17.00–20.00)^§^	20.00 (19.25–24.00)^§^	**<0.001**^c^

Bolded values indicate statistical significance.

^a^ P value based on independent sample t test

^b^ P value based on the chi-square test of independence

^c^ P value based on the Mann-Whitney U test

* Mean±SD

^§^ median (IQR, Q1-Q3)

### Binary logistic regression analysis of patient’s loss to follow-up in long-term supportive periodontal therapy

Table 3 shows The sub-variable for multinomial variables was set in SPSS. Table 4 shows the results of binary logistic regression analysis of patient’s loss to follow-up in long-term supportive periodontal therapy. The adjusted odds ratio of age was 1.042 (95% CI: 1.012–1.074). The adjusted odds ratio of failing to enter into supportive periodontal therapy for moderate periodontitis compared with severe periodontitis was 4.892 (95% CI: 2.280–10.499). The adjusted odds ratio of failing to enter into supportive periodontal therapy for non-surgery compared with periodontal surgery was11.334 (95% CI: 2.235–57.472). The adjusted odds ratio of failing to enter into supportive periodontal therapy for the middle-scoring group (54–59) compared with the high-scoring SESS group (60–75) was 4.800 (95% CI: 2.263–10.182) and 71.899 (95% CI: 23.926–216.062) for the low-scoring group (15–53). A significant association was observed between age, severity of periodontitis, SESS scores and failing to enter into supportive periodontal therapy in the crude analysis.

**Table 3:** Categorical variable encoding.

Variable	Factor	Assignments
X1	Severity of periodontitis	Moderate = 1 Severe = 0
X2	Periodontal surgery or not	non-surgical = 1 surgical = 0
X3	SESS score group	Low (1,0) middle (0,1) high (0,0)
X4	Education level	Baccalaureate education and higher = 0 Associate degree education and lower = 1
Y	Loss to follow-up or not	Not loss = 0 loss = 1

**Table 4:** Binary logistic regression analysis of loss to follow-up in long-term supportive periodontal therapy.

Item	Category	*β*	*SE*	*Wald*	*Sig*.	*Exp(B)*	*95%CI*
Age		0.042	0.15	7.642	**0.006**	1.042	1.012~1.074
Education level	Associate degree education and lower	-0.314	0.489	0.411	0.521	1.368	0.525~3.569
	Baccalaureate education and higher	-	-	-	-	1.000	-
Severity of periodontitis	moderate	1.588	0.390	16.615	**<0.001**	4.893	2.280~10.499
	severe	-	-	-	-	1.000	-
Periodontal surgery	no surgery	2.428	0.828	8.591	**0.003**	11.334	2.235~57.472
	surgery	-	-	-	-	1.000	-
SESS score group	low	4.275	0.561	57.996	**<0.001**	71.899	23.926~216.062
	middle	1.569	0.384	16.722	**<0.001**	4.800	2.263~10.182
	high	-	-	-	-	1.000	-

Bolded values indicate statistical significance.

## Discussion

This study revealed significant difference between China and developed countries in the proportion of patients entering into supportive periodontal therapy. Several studies in developed countries reported a higher proportion of patients entering into supportive periodontal therapy than the proportion who fail to enter into supportive periodontal therapy in long-term periodontal therapy[9,16,24,25]. However, our study demonstrated an inconsistent result. A higher proportion of patients did not enter into supportive periodontal therapy than those who entered into supportive periodontal therapy, which indicates that the compliance of patients entering into supportive periodontal therapy was somewhat unsatisfactory. Chinese clinical oral development was initiated relatively late compared with developed countries. Awareness of oral self-care was weak in the Chinese population. The appearance of obvious oral pain symptoms was the primary reason for consulting with a dentist[26]. The popularity of gargling dates back to ancient China, and tooth brushing was considered impolite[27]. Tooth brushing was not accepted until the 1970s[28]. Oral health examination was subordinated to other health check-up items in China in recent years, and the oral cavity project was sidelined from basic project expert consensus on physical examination in 2014[29]. Yang[30] reported that oral health examination was provided at her research site, which is a physical examination center in a tertiary care hospital in Xi’an, China. Oral health self-care has gained powerful financial support in China, but a large gap in the consciousness of oral health self-care remains between China and many developed countries.

A higher level of oral hygiene-related self-efficacy was found in Germanic patients with periodontal disease[9]. Our study demonstrated that the SESS scores of patients with chronic periodontitis was at a middle level, which is consistent with Kakudate[16]. However, these two studies were quite different in the severity of periodontitis and the location of research sites. Kakudate[16] focused on patients with mild to moderate chronic periodontitis in a private dental clinic, and we recruited the patients with moderate to severe chronic periodontitis in a tertiary care hospital. The reason for our selection was that patients with moderate to severe chronic periodontitis represent most periodontitis patients in China. Patients with chronic periodontitis did not consult with dentists until the condition was very serious. These results further confirmed that the Chinese population exhibited lower oral health awareness compared with developed countries. The effectiveness of traditional dental health instructions on Chinese patients was not sufficient. Therefore, an effective intervention method that is appropriate for Chinese people should be further examined.

Woelber demonstrated that oral hygiene-related self-efficacy was a key factor in oral health behavior and effectively predicted patient oral health outcome in Germany[9]. Kakudate et al. viewed self-efficacy as an indicator of loss to follow-up for short- and long-term periodontal treatment in Japan[7,16]. Our study found that patients with higher SESS scores were more likely to enter into supportive periodontal therapy after completion of non-surgical treatment. We considered SESS as a useful tool to predict all possibilities for a patient’s failure to enter into supportive periodontal therapy in Tianjin, China. Oral self-care self-efficacy should be improved and constantly strengthened in the process of oral health education.

We observed that in China, older patients were more likely to fail to enter into supportive periodontal therapy than younger patients were, which is inconsistent with the previous studies that found that patients who were older tended to remain in periodontal treatment[7,16,31,32]. We also analyzed the association between periodontitis severity and loss to follow-up in long-term supportive periodontal therapy and noted a positive predictive effect on long-term supportive periodontal therapy. Patients with severe periodontitis tended to exhibit better compliance with entry into supportive periodontal therapy. Alogna found that compliant patients exhibited significantly more severe periodontitis than non-compliant patients, who tended to exhibit more of an internal locus of control[33]. We inferred that patients with severe chronic periodontitis were more aware of the seriousness of their disease situation and paid more attention to oral self-care. Graetz reported that patients who had a surgery were more compliant than patients without surgery[34], which was consistent with our findings.

No significant relationship was observed between compliance and gender, which is consistent with earlier reports[35–37]. The present study observed that gender was not significantly different between the two groups, which is supported by previous studies[7,14]. Kakudate[7] found a deeper probing depth at patients’ initial visit was associated with a greater tendency to enter into supportive periodontal therapy, which is inconsistent with the results of KÖnig[38]. Our study observed a positive trend in entry into supportive periodontal therapy and probing depth, but no statistical significance was found. We also analyzed the association between clinical attachment loss and loss to follow up in long-term periodontal therapy, and no significant relationship was observed. A previous study classified patients localized and generalized chronic periodontal disease[39]. One possible explanation for these results may be that the mean of probing depth and clinical attachment loss failed to define the severity of periodontal disease, which did not predict entry into supportive periodontal therapy in patients with chronic periodontal disease. Previous studies reported associations between several variables and the compliance of patients with periodontitis, except educational level and socioeconomic status[7,14]. We investigated whether educational level and monthly family income predicted the outcome of entry into supportive periodontal therapy. However, no significant difference was found between educational level, monthly family income and entry into long-term supportive periodontal therapy in our present study. Bao investigated the factors that influenced the compliance of periodontitis patients and observed that a patient’s cognitive level of periodontitis was the most important factor affecting compliance, but educational background and income had little effect on compliance[17]. Whether monthly family income affects compliance is not clear. One possible reason for this discrepancy is that most of the subjects in our study enjoyed resident medical insurance, which covers 55% of the total expenses. The expense for treating periodontitis is also relatively low compared to treatment for treating other diseases. Therefore, there is not much concern of the economic burden of long-term periodontal treatment.

### Limitations and future research

There are several limitations to this study that should be acknowledged. First, numerous variables are used to diagnosis periodontitis[40], and the present definition of periodontal disease may overestimate the severity. Second, the recruited subjects were patients with moderate and severe chronic periodontitis, but we did not examine the loss to follow-up in long-term supportive periodontal therapy in patients with mild chronic periodontitis. Kakudate previously reported that subjects with mild and moderate chronic periodontal disease exhibited higher SESS scores and were more likely to remain in periodontal treatment[7]. Patients with mild chronic periodontitis should be considered in future studies. Finally, we recruited only patients with chronic periodontitis in one stomatological tertiary care hospital, and other stomatological tertiary care hospitals and private dental clinics were not involved in Tianjin, China. Further large-sample multicenter studies in other institutions and clinics should be performed to verify our results.

## Conclusion

These findings suggest that age, periodontitis severity, periodontal surgery and the level of self-efficacy for self-care may be effective predictors of loss to follow-up in long-term supportive periodontal therapy in patients with moderate to severe chronic periodontitis. Older patients with more severe periodontitis and lower self-efficacy for self-care were more easily lost. The screening of patients who tend to become lost in long-term supportive periodontal therapy during an early visit would allow the dental staff to provide targeted and effective support to reduce the number of patients lost to follow-up in long-term periodontal treatment and promote the periodontal health of the patients with periodontitis.

## Supporting information

S1 File Outcome of logistic regression. (ZIP)
